# From Basic Visual Science to Neurodevelopmental Disorders: The Voyage of Environmental Enrichment-Like Stimulation

**DOI:** 10.1155/2019/5653180

**Published:** 2019-05-06

**Authors:** Alan Consorti, Gabriele Sansevero, Claudia Torelli, Nicoletta Berardi, Alessandro Sale

**Affiliations:** ^1^University of Pisa, Pisa, Italy; ^2^IRCCS Stella Maris, Calambrone, Pisa, Italy; ^3^Neuroscience Institute, National Research Council (CNR), Pisa, Italy; ^4^Department of Neuroscience, Psychology, Drug Research and Child Health NEUROFARBA, University of Florence, Florence, Italy

## Abstract

Genes and environmental stimuli cooperate in the regulation of brain development and formation of the adult neuronal architecture. Genetic alterations or exposure to perturbing environmental conditions, therefore, can lead to altered neural processes associated with neurodevelopmental disorders and brain disabilities. In this context, environmental enrichment emerged as a promising and noninvasive experimental treatment for favoring recovery of cognitive and sensory functions in different neurodevelopmental disorders. The aim of this review is to depict, mainly through the much explicative examples of amblyopia, Down syndrome, and Rett syndrome, the increasing interest in the potentialities and applications of enriched environment-like protocols in the field of neurodevelopmental disorders and the understanding of the molecular mechanisms underlying the beneficial effects of these protocols, which might lead to development of pharmacological interventions.

## 1. Introduction

The brain capability to adapt in response to environmental changes is called neural plasticity, which allows cerebral circuits to modify their structure and function in response to experience through changes occurring at the molecular, neuronal, and systemic level.

In all mammal species studied so far, major plastic changes are mostly confined to specific time windows, early in development, known as critical periods (CPs) [1, 2]. During these periods, different for distinct developing functions, the inner genetic plan and the external environmental influences cooperate, leading to the final unfolding and maturation of an adaptive individual body. At the end of CPs, neural plasticity levels decay, possibly as the result of evolutionary pressures towards a final stabilization and maintenance of the mature structural connections and of the ensuing sensory functions emerging from the developmental events.

A key consequence of the interplay between genes and environment underlying brain development is that genetic alterations and/or exposure to altered environmental conditions before the closure of CPs can lead to alterations of brain development, resulting in a number of different, moderate to severe, neurodevelopmental disorders [3, 4].

During the last decades, an increasing number of experimental researches have led to the discovery of molecular brakes that restrict neural plasticity within the temporal limits of the CPs [5–8]. The opportunity to regulate these molecules and to modulate the time course and closure of CPs have opened the possibility to ameliorate brain functioning in neurodevelopmental disorders even past the end of the CPs. In this context, the visual system emerges as a favorite model to probe cortical plasticity throughout and after the end of CPs, both in physiological and pathological conditions [9]. Indeed, since the original discovery by the Nobel Prize winners Wiesel and Hubel demonstrating the existence of a CP for ocular dominance plasticity in mammals with binocular sight [10], the visual cortex has become the most widely employed system to investigate the mechanisms underlying cerebral plasticity and the possibility to restore or enhance it in adulthood. Beyond its impact on the treatment of neurodevelopmental visual disorders such as amblyopia [11], this seminal work has opened new perspectives in the field of neurodevelopmental disorders which are not considered, in their essential nature, visual ones, such as Rett syndrome (RTT), autism spectrum disorders (ASD; in particular X-fragile syndrome (FXS)), and Down syndrome (DS) [12–16].

In particular, the study of the mechanisms underlying visual system plasticity in animal models and the specific impact that EE exerts on them has provided insights for the development of possible pharmacological and nonpharmacological [17–19] interventions in human subjects with RTT, DS, and FXS. In some occasions, these applications have already moved forward to the phase 3 of clinical experimentation or randomized studies [17–19].

In this review, we shall discuss the translational route from basic studies focused on visual system plasticity to the application of possible EE interventions in human subjects. Wherever possible, we shall underscore the relevance of a better knowledge of the molecular mechanisms underlying the EE effects in animal models for the characterization of similar mechanisms underlying neural dysfunctions in humans and for the development of possible successful interventions.

## 2. Manipulating the Environment to Enhance Plasticity: The Environmental Enrichment Approach

The most direct approach to manipulate the environment in order to enhance neural plasticity is environmental enrichment (EE), introduced in the early 1960s by Rosenzweig and colleagues [20–22]. EE consists in rearing laboratory animals in cages wider and more attractive than those employed in the so-called standard conditions (SCs), with a variety of sensory, cognitive, motor, and social stimuli. Exposure to EE exerts profound effects on brain morphology and physiology, enhancing neural plasticity in different brain areas at all ages analyzed so far (for review, see [23–25]) and exerting beneficial effects in animal models of neurodegenerative diseases and brain injury [26].

The definition of EE is based on a comparison with a reference condition that, for laboratory animal models, is generally represented by SCs, in which the animals are reared in simple cages without any other object than litter, food, and water, and are hosted in very small social groups. Thus, one critical question is to what extent is the EE approach able to provide supernormal levels of stimulation or whether it should be better considered a way to compensate for sensory-motor deprivation associated with SCs. According to this criticism, the beneficial results obtained with EE in animal models might be of reduced interest in terms of their applicability to the clinic, as humans are generally considered already “enriched” in their living conditions (see also [27]). As originally stated by the first proposers of the EE approach, it is worth considering that, after hundreds of generations in SCs, a strong genetic drift with respect to wild natural populations may have rescaled neural development and basic brain functions in a new physiological and well-adapted dimension, without any pathological or aberrant side effect for brain development. Thus, measures collected in these simplified models may actually represent a suitable source for normative data, to be compared with the effects deriving from exposure to EE.

## 3. When Experience Affects Development: The Case of Amblyopia

An unbalanced stimulation of the two eyes during early postnatal development induced by variable causes such as congenital cataract, unequal refractive power, or strabismus can lead to a neurodevelopmental visual deficit known as amblyopia (lazy eye). This disease has an incidence of 1-5% in the worldwide population, and it is the most prevalent one-eye visual impairment, characterized by a loss in visual acuity, low contrast sensitivity, hampered stereopsis, and an impairment of the orientation tuning of cortical neurons (binocular matching) [28–30]. Amblyopia is considered a purely cortical deficit with no detectable impairments in peripheral regions, albeit the lateral geniculate nucleus may be anatomically and functionally involved [24, 31]. A timely patching of the spared eye performed during the CP for binocular vision and visual acuity development (approximately until 8 years of age in humans) is normally associated with a rescue from amblyopia. Nevertheless, the closure of CP turns amblyopia into an almost untreatable disease.

Amblyopia is easily modeled in animals, keeping one eye deprived of pattern vision via prolonged eyelid suture (monocular deprivation (MD)), started during the CP and protracted until adulthood [32, 33]. The procedure causes a marked ocular dominance shift towards the open eye in the binocular neurons of the primary visual cortex, determined by functional and structural empowering of the inputs emerging from the ipsilateral/spared eye, at the expense of those from the contralateral/deprived one [34].

In recent years, EE has proven successful in the treatment of amblyopia in adult animals. Adult amblyopic rats that were transferred to an EE setting for three weeks displayed a full recovery of visual acuity, ocular dominance, and depth perception [7, 35]. More selective EE conditions are also able to reproduce the beneficial effects elicited by the entire complex enriched experience, especially when motor or visual stimuli are specifically enhanced [36]. In particular, three weeks of voluntary physical exercise induced a full recovery of visual acuity and ocular dominance in adult amblyopic rats [36]. Also data from Stryker's lab confirmed the potential of motor activity as a booster of visual responsiveness and plasticity in the visual cortex, showing that running on a treadmill enhances visual cortical activity in mice [37] and promotes visual function recovery following monocular deprivation [38]. Another condition akin to EE, i.e., practicing in a two-choice active visual discrimination task, also resulted in an almost-full rescue of visual acuity and ocular dominance in adult amblyopic animals [36, 39].

Animal model data provided also information on the mechanisms underlying EE-like effects in promoting recovery from amblyopia. Data from Stryker's lab showed that enhancement of visual cortical activity [37] and visual function recovery following monocular deprivation [38] in running mice is associated with a disynaptic disinhibition involving activation of VIP+ interneurons and inhibition of SOM+ interneurons in the visual cortex [40]. Our work and other labs showed that exposure to EE reduces GABAergic inhibition in the visual cortex of enriched animals [7, 41]. Recovery of visual functions in enriched amblyopic rats was accompanied by increased expression of BDNF, reduction in the intracortical inhibition-excitation balance, and reduced density of perineuronal nets made by chondroitin sulphate proteoglycans enwrapping the terminals of GABAergic interneurons [7, 15]. Moreover, exposure to EE increased levels of serotonin in the adult visual cortex and a pharmacological blockade of this enhancement prevented EE-dependent restoration of visual cortex plasticity in adult animals [15]. Interestingly, both motor activity and PL also led to a reduced synaptic release of GABA in the visual cortex of adult amblyopic rats [36].

EE and physical exercise also contribute to increase insulin-like growth factor-1 (IGF-1) in the brain. IGF-1 has a crucial role in setting the pace of visual development and seems to be a “master mediator” of EE effects, upstream of BDNF, correcting, for instance, the mismatch between two visual developmental processes, ocular dominance development, and binocular matching of orientation selectivity development, caused by genetic overexpression of BDNF [42, 43]. This is important to underline, since the possibility that different molecules or the same molecule but in different neurons can differently affect developmental trajectories and functional recovery is now suggested not only by the effects of BDNF on binocular matching but also by Ngr1 deletion on visual acuity and ocular dominance recovery in amblyopic mice [31]. Confirming its nature of master experience mediator, the administration of IGF-1 in the adult visual cortex promoted recovery of visual acuity and ocular dominance in adult amblyopic rats, an effect paralleled by the reduction of intracortical GABA levels [44].

Given its noninvasive nature, the concept of EE appears as a promising strategy to counteract visual impairments in human amblyopia. The major challenge is how to transfer EE to human life conditions, setting up the best protocols to induce a suitable environmental stimulation for human patients. Recent papers show very encouraging data. Active videogames appear a clever trick to combine key EE components such as visual attention and enhanced sensory stimulation (see [45]), with promising results in adult subjects with amblyopia [46], but with apparently limited effects in children [47, 48]. In the same context, engagement in subtle visual discrimination tasks such as those associated with visual perceptual learning (see [39] for a recent review) can favor recovery of visual functions in adult amblyopia (e.g., [49–60]). Very recently, moderate levels of voluntary physical activity combined with short-term monocular deprivation have been shown to enhance homeostatic plasticity in the visual cortex of healthy human subjects, favoring the dominance of the briefly deprived eye [61]. Most importantly, brief occlusion of the amblyopic eye combined with enhanced physical activity promoted a remarkable and long-lasting recovery of visual acuity and stereopsis in adult amblyopic individuals [62].

Thus, basic studies on the impact of EE on visual system plasticity are currently leading to an increasing interest for the development of promising nonpharmacological interventions in amblyopic human subjects. Future research should try to provide evidence on the effectiveness of such active training on amblyopia recovery in different categories of human amblyopic subjects and to ascertain whether the documented beneficial effects in humans are due to the same mechanisms already verified in animal models.

## 4. When Genes Affect Development: The Case of Down Syndrome and Rett Syndrome

Differently from amblyopia, Down syndrome (DS) and Rett syndrome (RTT) are developmental disorders of genetic nature. Originally described by John Langdon Down, DS is the most widespread genetic form of intellectual disability and it is caused by the total or partial triplication in the genome of the chromosome 21 [63]. This has a dramatic impact on the central nervous system, with a disruption of the synaptic architectures leading to a failure in cognition, learning, memory, and language [64, 65]. The genetic imbalance does also result in severe consequences in extracognitive domains, such as in the visual system, with damaged spatial acuity and increased incidence of strabismus and cataract [66]. Moreover, since the gene encoding the amyloid precursor protein (APP) is located on the chromosome 21, trisomy induces an increase in the concentration of brain *β*-amyloid, and adult DS individuals of more than 40 years of age display early-onset Alzheimer-like neuropathology that additionally complicates their quality of life and independence possibilities [67, 68].

The complexity of the DS made its replication in animal models a highly demanding aim. Generated in the 1990 [69], the Ts65Dn mouse represents the most commonly used model to study this pathology. Ts65Dn mice bear a segmental triplication of the chromosome 16 that displays high degree of synteny with the human chromosome 21 [70]. The resulting mutation closely resembles the structural and behavioral features of the human disorder. Ts65Dn mice display decreased long-term hippocampal potentiation, defective neurogenesis, low synaptogenesis, and a generalized state of cerebral overactivation of GABAergic circuits [65, 71]. Remarkably, similarly to human subjects with DS, trisomic mice display severe visual deficits: the visual acuity is significantly impaired, visual evoked potentials are slower than normal, and the visual cortex responsiveness is anomalously shifted towards the ipsilateral inputs [72, 73].

RTT is a debilitating progressive disorder first noted by Andreas Rett in 1966 [74]. It is a rare pathology affecting quite exclusively females, with an incidence of about 1 over 10,000 births. With very few exceptions [75], the majority of males with RTT die soon after delivery. RTT remains mostly asymptomatic during the first months of postnatal growth. Thereafter, most of the skills already acquired by an affected subject dramatically deteriorate. As RTT lacks a specific cortical localization, deficits involve the whole brain functionality, with some prototypical characteristics including severe motor deficits (stereotyped hand movements are the principal RTT hallmark), autonomic dysfunctions, and intellectual disability [76, 77]. Only in 1999 [78], these deficits were first associated to loss-of-function mutations in the gene encoding the methyl-CpG-binding protein (MeCP2) on the X chromosome, thus clarifying discrepancy in the incidence between females and males. The MeCP2 protein has a proven role as a master regulator of the chromatin state and gene expression (including the *BDNF* gene [79]), being involved in the formation of a multiprotein complex that binds methylated CpG regions and allows gene silencing [80]. Recent evidence expanded this view, suggesting that it could also activate the expression of several other genes, playing as an activator or a repressor depending on the type of proteins that join the complex [81]. The deletion of the MeCP2 gene in mouse models leads to a phenotype that closely recapitulates many features of the human disorder [82]; thus, employment of mouse models has become essential to study the mechanisms involved in RTT and to test the potential useful treatments. A recent paper documented, in girls with RTT, visual deficits similar to those found in Mecp2 heterozygous female mice, and underscored the possibility to successfully exploit visual evoked potentials (VEPs) as an unbiased, quantitative biomarker to monitor brain function in RTT [13].

Strikingly, the EE approach turned out to be very valuable in the context of these genetic disorders [83–86]. Exposure of either developing or adult Ts65Dn mice to EE induces a marked recovery of both cognitive and visual functions [72, 87], and middle aged Ts65Dn mice chronically maintained in EE conditions displayed a reduced amount of *β*-amyloid oligomers compared to trisomic mice reared in SCs [88]. In *Mecp2*mutant mice, EE ameliorated motor coordination and motor learning and rescued memory deficits and anxiety-related behavior, with gender differences [89].

As seen for visual disorders, physical exercise emerges as one critical component underlying the beneficial EE effects for DS, being specifically associated with an increased neurogenesis and gliogenesis in the hippocampus [90, 91]. Recently, the specific effect of physical exercise was also explored in the Mecp2(+/-) mouse model of RTT, with the demonstration that increased voluntary physical activity normalizes the physiology of the hypothalamic-pituitary-adrenal axis, providing a significant rescue from affective behavioral dysfunctions [92].

The positive impact of EE on both DS and RTT has been linked, in animal models, to modulation of GABAergic synaptic strength and to an increased BDNF expression [72, 87, 89, 93].

Based on the results obtained in animal models of DS and RTT, recent studies have started to apply the EE paradigm to infants and children with these disorders. Different kinds of early multisensory intervention have been associated with beneficial effects on the maturation of visual functions in infants with DS [19] and improved gross motor skills and increased blood BDNF levels in children with RTT [18].

Thus, as seen for amblyopia, a general picture emerges in which results obtained in animal models might orient future research in humans, with the aim to uncover shared molecular mechanisms that might be instrumental for the development of suitable pharmacological approaches.

## 5. Towards an Environment-Based Pharmacological Approach?

The remarkable capacity of the EE approach to trigger recovery in diseases as different as amblyopia or genetic intellectual disabilities could be due, at least in part, to its impact on the GABAergic circuitry. An increased activation of the GABAergic inhibitory system is widely considered as a common hallmark of many brain developmental pathologies [94, 95]. Unfortunately, availability of suitable therapeutic compounds that may safely act in decreasing the activation in the GABAergic system is scant, while most of the drugs have severe proconvulsive side effects, with consequent rejection by FDA.

In this context, fluoxetine, a selective serotonin reuptake inhibitor (SSRI) widely prescribed in the treatment of human depression, emerges as a potentially interesting candidate for drug repositioning, given its capability to increase levels and availability of serotonin, one key molecular factor underlying EE effects [15].

Adult amblyopic rats chronically treated with fluoxetine display robust recovery of visual cortex plasticity and visual functions, together with increased BDNF and a reduced GABAergic tone in the primary visual cortex [96]. A very recent study examined the effect of fluoxetine in adult amblyopic human subjects, without a significant improvement in visual performance compared to that obtained in subjects treated with placebo [97]. Since all patients did also perform, during the 10 weeks of pharmacological treatment, an intense perceptual training therapy, it remains unclear whether the lack of a specific effect of fluoxetine in this study was due to a ceiling effect of the training paradigm.

Administration of fluoxetine for eight weeks in the drinking water reduced brain GABA release and rescued hippocampal synaptic plasticity and spatial memory in DS mice [98]. Moreover, treating neonate Ts65Dn mice with fluoxetine led to a full recovery of dentate gyrus neurogenesis and hippocampus-dependent memory performance [99]. Based on these results, the effectiveness of fluoxetine in human subjects with DS is, at the moment, under evaluation in several clinical trials [100, 101].

It remains unclear whether the therapeutic effects of fluoxetine are due to its action on the GABAergic system or are also dependent on its recognized ability to increase BDNF levels [102, 103]. BDNF itself, indeed, could emerge as a helpful compound to treat amblyopia and genetic disorders like DS and RTT. The promising potential of BDNF, however, is thwarted by the impossibility for this neurotrophic factor to efficiently cross the blood-brain barrier when delivered via peripheral administration [104]. Recently, intranasal BDNF administration, a safe procedure considered quite effective to target proteins to the central nervous system [105], induced recovery of visual acuity, ocular dominance, and visual depth perception in adult amblyopic rats. Moreover, the administration of 7,8-dihydroxyflavone, an agonist of the BDNF receptor TrkB, efficiently restored learning and memory abilities in Ts65Dn mice [106]. In heterozygous female *Mecp2* mutant mice, pharmacologic activation of the BDNF receptor TrkB ameliorated several biochemical and functional abnormalities, highlighting TrkB as a possible therapeutic target in this disease [107].

Several papers showed that treatment with either a fragment of IGF-1 or the full-length molecule can be effective in alleviating symptoms in RTT mouse models (reviewed in [108]). Based on these studies, the application of IGF-1 to RTT patients has recently started (e.g., [109, 110]).

**Figure 1:**
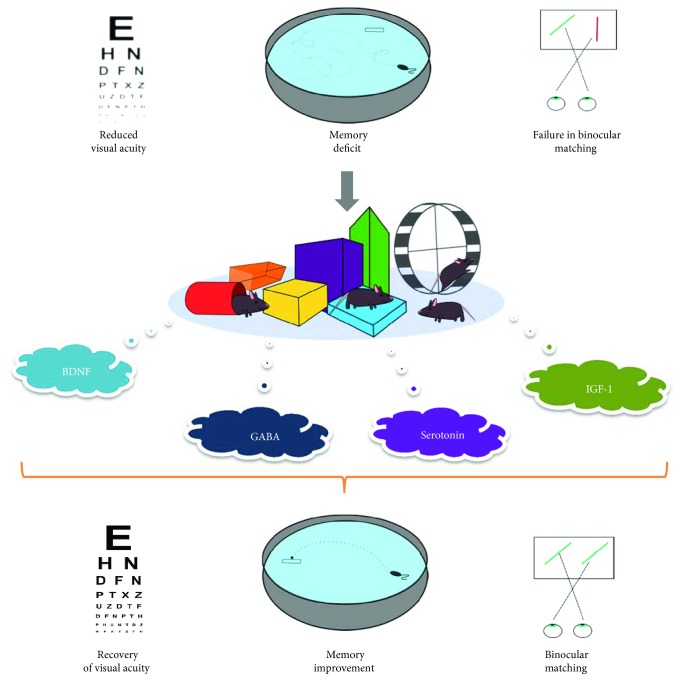
Exposure to conditions of environmental enrichment modulates a number of key molecular factors involved in brain plasticity and repair, favoring recovery of sensory functions (e.g., visual acuity and binocular matching) and improvement of learning/memory abilities in neurodevelopmental disorders. The molecular factors involved in the beneficial effects elicited by enrichment-like conditions can become the target for successful pharmacological manipulations and potential translational application to the clinic.

In conclusion, combining EE with classical studies on visual system plasticity has led to the characterization of several potential molecular targets for successful translational applications (Figure 1). The therapeutic value of the emerging molecular pathways overcomes the boundaries of the visual system and opens the way for further testing in the treatment of several neurodevelopmental disorders of different genetic or environmental origin [104, 111–114]. Future studies should exploit the EE approach in animal models (applied either as a multicomponent or as a channel-specific strategy) as a source for translational application to human patients. Knowledge about shared molecular pathways might inspire the development of new pharmacological strategies for still cureless developmental disorders.
